# Translating 2D Director Profile to 3D Topography in a Liquid Crystal Polymer

**DOI:** 10.1002/advs.202004749

**Published:** 2021-02-24

**Authors:** Pengrong Lv, Yuxin You, Junyu Li, Yang Zhang, Dirk J. Broer, Jiawen Chen, Guofu Zhou, Wei Zhao, Danqing Liu

**Affiliations:** ^1^ SCNU‐TUE Joint Lab of Device Integrated Responsive Materials (DIRM) National Center for International Research on Green Optoelectronics South China Normal University No 378, West Waihuan Road, Guangzhou Higher Education Mega Center Guangzhou 510006 China; ^2^ Molecular Materials and Nanosystems and Institute of Complex Molecular Systems Eindhoven University of Technology P.O. Box 513 Eindhoven 5600 MB The Netherlands; ^3^ Solar Energy Research Institute Yunnan Normal University Kunming 650500 China; ^4^ Institute for Complex Molecular Systems Eindhoven University of Technology Den Dolech 2 Eindhoven 5612 AZ The Netherlands; ^5^ Department of Chemical Engineering and Chemistry Eindhoven University of Technology Den Dolech 2 Eindhoven 5612 AZ The Netherlands; ^6^ Guangdong Provincial Key Laboratory of Optical Information Materials and Technology & Institute of Electronic Paper Displays South China Academy of Advanced Optoelectronics South China Normal University Guangzhou 510006 P. R. China; ^7^ Shenzhen Guohua Optoelectronics Tech. Co. Ltd. Shenzhen 518110 China

**Keywords:** dielectric structuring, dynamic surface topographies, imprinted polymer flow pattern, liquid crystal polymer

## Abstract

Morphological properties of surfaces play a key role in natural and man‐made objects. The development of robust methods to fabricate micro/nano surface structures has been a long pursuit. Herein, an approach based on molecular self‐assembling of liquid crystal polymers (LCPs) is presented to directly translate 2D molecular director profiles obtained by a photoalignment procedure into 3D topographies, without involving further multi‐step lithographic processes. The principle of surface deformation from a flat morphology into complex topographies is based on the coupling between electrostatic interactions and the anisotropic flow in LCPs. When activated by an electric field, the LCP melts and is driven by electrohydrodynamic instabilities to connect the electrode plates of a capacitor, inducing topographies governed by the director profile of the LCP. Upon switching off the electric field, the formed structures vitrify as the temperature decreases below the glass transition. When heated, the process is reversible as the formed topographies disappear. By pre‐programming the molecular director a variety of structures could be made with increasing complexity. The height, pitch, and the aspect ratio of the textures are further regulated by the conditions of the applied electric field. The proposed approach will open new opportunities for optical and electrical applications.

## Graphical abstract

A technique that stores latent information that can be retrieved repeatedly by subjecting the film to an electrical field is presented. Under the electric field, surfaces morph reversibly from flat to a well‐defined corrugated state, while maintaining its layered molecular organization. Anisotropic polymer flow is generated for this purpose using dielectric energy and directed by an underlying 2D structured monolayer.

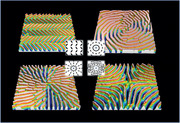

## 1 Introduction

Morphology, especially at the surface, is a vital surviving strategy for living creatures in nature. Their cleverness lies in translating microscopic molecular organizations into macroscopic shapes in response to changes in their environment. For example, mammals fluctuate their shapes or reflex piloerection at their skin to protect against predators and create insulation in cold condition.^[^ ^1^ ^]^ When in danger, chameleon changes its color by altering the arrays of the structures in the epidermis.^[^ ^2^ ^]^ Inspired by nature, scientists have developed several technologies to fabricate surface patterns,^[^ ^3^ ^]^ most of them originate from conventional lithographic procedures, including photo‐embossing,^[^ ^4^ ^]^ soft‐lithography,^[^ ^5^ ^]^ and micro‐machining. The use of photonic processes is convenient as they represent mature technologies and are well developed for the semiconductor industry. But they have the disadvantage that complex and expensive optics are required and tedious fabrication steps are involved. Occasionally, electricity‐induced structure formation is proposed.^[^ ^6^ ^]^ An interesting example is based on the material flow of an isotropic polymer driven by the electric field.^[^ ^7^ ^]^ Although lithography is not directly involved in the structure formation process, patterned electrodes are necessary to guide the polymer flow. Inspired by this method, in this work, we propose an approach to create surface protrusions from a flat polymer coating by a non‐patterned electric field. The underlying mechanism is based on generating anisotropic material flow in aligned liquid crystal polymers (LCPs) as set by their directional dependent viscosity. Under the electric field provided by two continuous electrodes, LCP migrates unidirectionally and grows into surface textures with regular periodicity. This process continues to progress until the LCP protrusions reach the opposite electrode.

LCPs are known for their anisotropic properties. Their large optical and dielectric anisotropy was intensively studied for electro‐optical devices,^[^ ^8^ ^]^ such as, displays and smart windows.^[^ ^9^ ^]^ Recently, their anisotropic mechanical properties have attracted considerable attention in the field of actuators and soft robotics.^[^ ^10^, ^11^, ^12^, ^13^, ^14^, ^15^ ^]^ For instance, in freestanding LCP films, a variety of deformations have been demonstrated with capacities in grabbing objects,^[^ ^16^, ^17^ ^]^ lifting weights,^[^ ^18^ ^]^ and transporting materials. And morphing of an LCP coating surface, ranging from regular topographic stripes^[^ ^19^, ^20^, ^21^ ^]^ to irregular fingerprint‐like textures, reach out to applications in controlled tribology, particles mitigation^[^ ^22^ ^]^ and cell growth.^[^ ^23^ ^]^ In their molten state, LCPs exhibit anisotropic flow properties which is well explored in injection molding of thin‐wall miniaturized objects, 3D/4D printing of actuators^[^ ^24^, ^25^ ^]^ and the formation of fiber arrays at interfaces.^[^ ^26^ ^]^

In this work, we explore the flow anisotropy of a side‐chain LCP to control the formation, size, and directionality of topographic structures that develop under electrohydrodynamic actuation. This approach has the advantage that the formed topographies have a significantly high aspect ratio of 0.5, in contrast to previous reports on electric dynamic surfaces in LCPs whose aspect ratio is ≈0.04.^[^ ^22^ ^]^ The major obstacle of the structures obtained via current technologies for application is the small‐sized height (<micrometer). While our proposed method enables the deformation amplitude to reach 6 µm, corresponding to 200% of the initial film thickness, from an exceedingly smooth surface. Moreover, it is a molecular self‐assembling process, which intrinsically gives the lateral dimension down to sub‐micrometer to micro‐meters range.^[^ ^27^ ^]^ The larger surface modulation originates from liquid crystal steered materials transport, while the previous principles are based on order parameter based stresses. We discovered that depending on the phase of the LCP, the surface patterns are formed either parallel or perpendicular to the director. We further demonstrate the versatility of this method that various complex 3D structures, such as, zigzag, radius, and azimuthal can be engineered.

## 2 Results and Discussion

**Figure 1:**
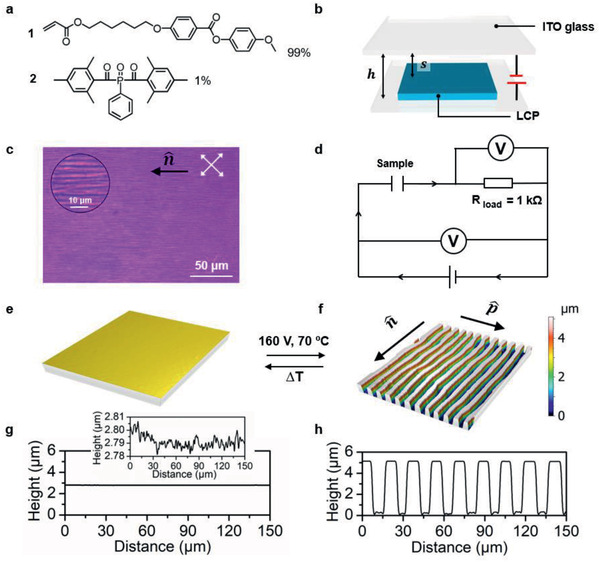
Principle of the formation of surface topographies. a) Materials used for electric‐induced anisotropic LCP flow. b) Device configuration. c) The initial coating as observed by optical microscopy between crossed polarizers. d) Equivalent electric circuit of the driving scheme. 3D profilometer measurements: e) 3D image of the initial flat surface and g) its corresponding 2D profile, expanded in height in the insert. f) 3D image of the actuated surface topographies after the actuation under a DC field and h) the corresponding 2D profile.

We prepared a side‐chain LCP film by in situ photopolymerization of an aligned liquid crystal mono‐acrylate **1** in its monotropic nematic phase in the presence of photoinitiator **2** (**Figure** **1**a). The alignment is controlled by a photoalignment layer beneath the monomer and is maintained during polymerization. The polymer exhibits a small negative dielectric anisotropy (Figure S1, Supporting Information). During the polymerization the polymer transition temperatures, as characterized by differential scanning calorimetry (Figure S2, Supporting Information), increase and a transition from the nematic to a smectic phase occurs.^[^ ^28^ ^]^ This smectic phase, which does not exist in the monomer, appears in the temperature range between 40 and 90 °C. Above this temperature, the LCP is nematic and becomes isotropic above 120 °C. We analyzed the mechanical properties of the polymer film by dynamic mechanical analysis (DMA) indicating a glass transition temperature (*T*_g_) at 46 °C (Figure S3, Supporting Information).

The device configuration we used for our further study is illustrated in Figure 1b, the uniaxial aligned LCP film with thickness *t* = 2.8 µm adheres to one electrode with a 10 nm photoalignment layer in between. A counter‐electrode is mounted at a distance *h*. The spacing *s* between the LCP and the counter electrode is filled with air. Here, *h* is chosen as 6 µm. In a first series of experiments, the photoalignment layer at the bottom ITO glass was exposed by a digital mirror device (DMD) to align the LC monomer homogeneously and uniaxially parallel to the substrate. The top ITO glass plate is plain without the presence of an alignment layer. To enhance flow property, the LCP coating is brought at an elevated temperature, well above its *T*_g_. In this method, the molecular alignment is an overarching factor. We first exam the formation of protrusions in the smectic, nematic, and isotropic phase of the polymer by actuating the coating at 70, 93, 130 °C, respectively (Figure S4, Supporting Information). Results suggest that smectic LCP exhibits the finest structures. While in nematic LCP, the formed line textures are less defined. Surprisingly the alignment of the line‐shaped protrusions is parallel to the initial director for smectic polymer and orthogonal for the nematic polymer, which we will explain by preferential flowability (vide infra). At the isotropic state, flow directionality is diminished. A comparison between the structures formed in the various phase can be found in Figure S4, Supporting Information. Based on these observations, we choose for smectic LCP in this work.

**Figure 2:**
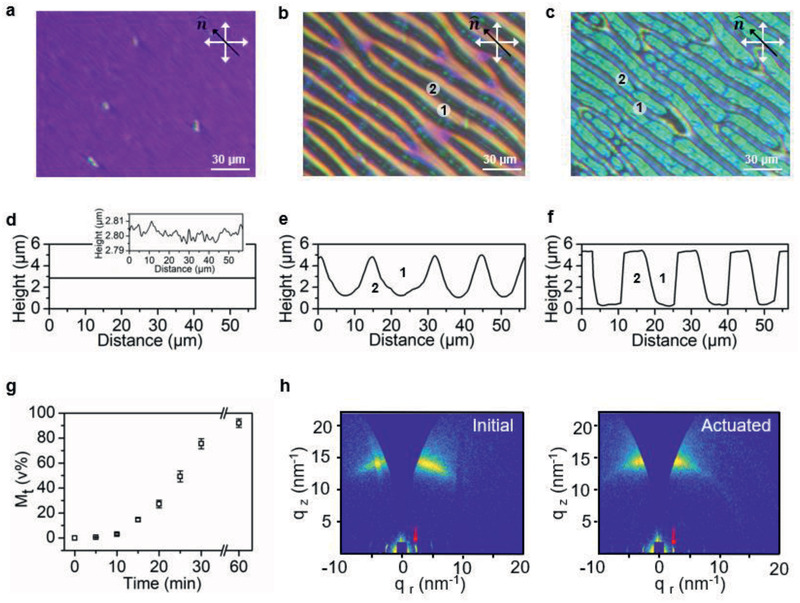
Progress of protrusion formation. a–c) Polarized optical microscopic images show the formation of stripe textures and the corresponding changes in the birefringent color. The pictures are the snapshot of the Movie S1, Supporting Information. d–f) Corresponding surface profiles of (a–c) measured by 3D profilometer. Insert in (d) is the zoomed‐in surface profile. g) Material transport from region **1** to region **2** calculated in volume percentage. h) 2D GIWAXS measurements, from left to right, the initial coating in absence of an electric field and the actuated protrusions. The red arrow marks the location of the smectic peak.

A typical smectic texture prior to the electrical actuation is presented in Figure 1c, which is also confirmed by 2D grazing‐incidence wide‐angle X‐ray scattering (GIWAXS) (**Figure** **2**h, left). The peaks at 2.6 nm^−1^ are attributed to the smectic periodicity of the LCP photo‐polymerized by molecular 1. The uniaxial orientation of the LCP is also confirmed by polarized optical microscopy (POM) showing birefringent purple color between the crossed polarizers with the director at 45^o^ and a black state when the director was parallel to one of the polarizers. In addition to the long‐range molecular director $\hat{n}$ liquid crystals in their smectic phase are characterized by the translational ordering of the molecules into layers defined by layer vector $\hat{p}$. The morphology of the coating surface is characterized by 3D profilometer. Initially, in the absence of the electric field, the LCP film exhibits a close to flat surfaces with minor undulations of tens of nanometers (Figure 1g). When this initially flat film is subjected to a direct current (DC) electric field at 160 V, as connected with the electric circuit given in Figure 1d, the surface deforms and the stripe protrusions with their longitudinal axis parallel to the molecular director $\hat{n}\;$appear. A typical surface topography is presented in Figure 1f. In this specific example, the height of the formed structures is 5.3 µm, which is ≈200% of the initial thickness, while the structure width is 10 µm suggesting its aspect ratio is 0.5.

By switching off the electric field, the formed structures can be either maintained by quenching below *T*_g_ or removed through thermal relaxation by maintaining the temperature above *T*_g_ (Figure S5, Supporting Information).

The formation of protrusions in non‐ordered isotropic polymers has been described before, which stems from the balance between the electrostatic force and surface energy.^[^ ^7^ ^]^ The electrostatic force induced by the DC field brings instabilities in the initial flat polymer film, which drives the dielectric polymer melts to fill the gap between the bottom and top electrodes. While the surface tension, conversely, prohibits the surface undulations and stabilizes the polymer film. This energy balance instability leads to perturbations which subsequently increase by the in‐plane flow of material eventually growing into protrusions. Electrically‐stimulated instabilities have also been observed in low‐molecular weight liquid crystals.^[^ ^29^, ^30^, ^31^ ^]^ In that case the liquid surface is destabilized forming temporary undulations. To the best of our knowledge, it is the first time that such phenomenon is described in LCPs with the advantage that permanent 3D structures are being formed. In isotropic polymers, the flow is undirected and forms characteristic pillar patterns, which are also observed in the LCPs in its isotropic state (Figure S4c, Supporting Information). Here, by employing the LCP in its smectic state we introduce an anisotropy in its flow behavior. Smectic LCP melts exhibit anisotropy in their viscosity,^[^ ^32^, ^33^ ^]^ which because of the Frank elastic constants tend to be unidirectional with the lowest shear forces parallel to $\hat{p}\;$. This privilege flow orthogonal to the director $\hat{n}$ resulting in line shape structures with their longitude parallel to the molecular alignment. In nematic LCP, although less defined, the formed structures are orthogonal to $\hat{n}$ as polymer melt preferably transports along the average molecular orientation where the lowest viscosity is found (Figure S4b, Supporting Information). The schematic representation of the director during the process of structure formation is given in Figure S6, Supporting Information.

To elaborate further on the principles of their formation, we in situ monitored the evolution of the surface topographies under the influence of the electric field. We characterized this process by correlating the observation under POM (Figure 2a–c) with the surface deformations measured by 3D profilometer (Figure 2d–f). Upon actuation, stripe patterns start emerging, predominately from the initial defects and the coating surfaces transfer from the initial flat state to the protruded state. The height of the surface topographies is given in absolute value. Therefore, we deduce that the polymer melt flows from region **1** to region **2** with **2** forming a peak while **1** descending into a valley. The material migration manifests itself also under POM. The birefringent color in region **1** transits from purple to orange, while the line structures in region **2** appear dark with narrow green lines in the center. The darkening of region **2** is anticipated to come from the lens effect of the protrusions which in their intermediate states possess a curved top. The protrusions keep growing under the electric field till they touch the top electrode, which leaves the final surface profiles with a flat top. Consequently, the lens effect in region **2** diminishes and the birefringent green color reappears under the POM. The optical retardation colors in between the protrusions also change as the film becomes thinner, a process which can be monitored by the color changes of the Michael‐Lévy interference chart (Figure S7, Supporting Information). We calculated the volume ratio ***M***_t_ of the transported materials as the integral of the cross‐sectional area, over time. During this material transport, we keep the voltage across the device at 160 V. The result follows a curvilinear growth as plotted in Figure 2g. This trend is explained as with the progress of the protrusion growth the field strength increases at the air‐polymer film interface as the results of the reduction of the distance *s* (Figure 1b), which consequently accelerates the deformations. Eventually, when protrusions contact the top electrodes, ≈90 v% of the total material is accumulated in peaks, and the protrusion keeps growing in the width until most of the material between the protrusions is consumed. After deformation, the smectic conformation has been preserved with the order parameter being slightly increased as observed from Figure 2h.

**Figure 3:**
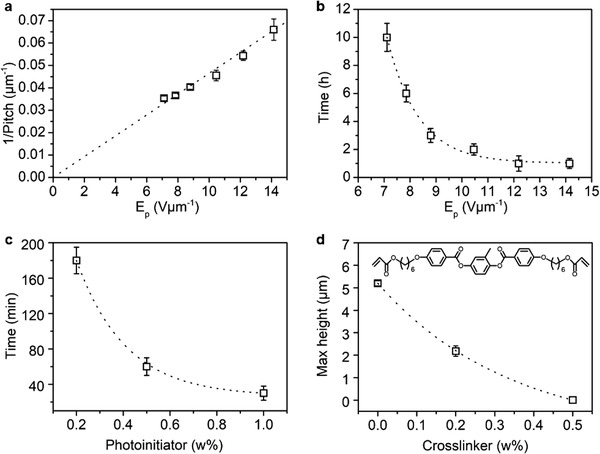
Parameters that influence the deformation. a) Pitch dependence on the applied electric field strength. b) The influence of voltage on the time required to reach deformation maxima. c) Increasing initiator decreases the time required for the polymer to reach maximum height of deformation. d) Crosslinking inhibits deformation seen by the reduction in maximum height of deformation as crosslinking is increased.

By modulating the electric field strength, we can adjust the pitch of the surface undulations and the deformation rate. The pitch is defined as the distance between two peaks of adjacent stripes. **Figure** **3**a shows that the pitch decreases with the increasing electric field. This is understandable as the higher electric field strength generates larger instabilities and more perturbations, which is also quantitative understood and the results are presented in the Figure S8, Supporting Information. In principle, the pitch varies from sub‐micrometers to infinite at zero voltage. In our experiments, we choose the conditions in which the pitch equal to 17 µm to determine the influence of the other parameters. One consequence of the increasing applied voltage is the decrease in time required to reach the maximum deformation as a larger electrostatic force is exerted (Figure 3b). Next, we investigated the influence of material properties on the deformation. We tuned the polymer viscosity by altering the molecular weight. It is known that photoinitiator concentration influences the polymerization characteristics. The presence of a higher photoinitiator concentration increases the generation of free radicals per unit of time, which induces competition in polymer chain growth. Consequently, as the amount of monomer in the system is limited the kinetic chain length remains shorter and the linear polymer has a lower molecular weight. In the experiment, we varied the concentration of photoinitiator from 0.2 to 1 w%, their corresponding molecular weight was measured by gel permeation chromatography and the results are given in Figure S9, Supporting Information. As elaborated in Figure 3c, the increase in the viscosity requires a longer time for the polymer melts to grow as caused by the reduction of mobility. We also slightly crosslinked the linear polymer by incorporating a diacrylate molecule (Figure 3d, insert). It is clear that already at a low concentration of 0.5 w% crosslinker, the flow is prohibited, and no measurable protrusions are created.

**Figure 4:**
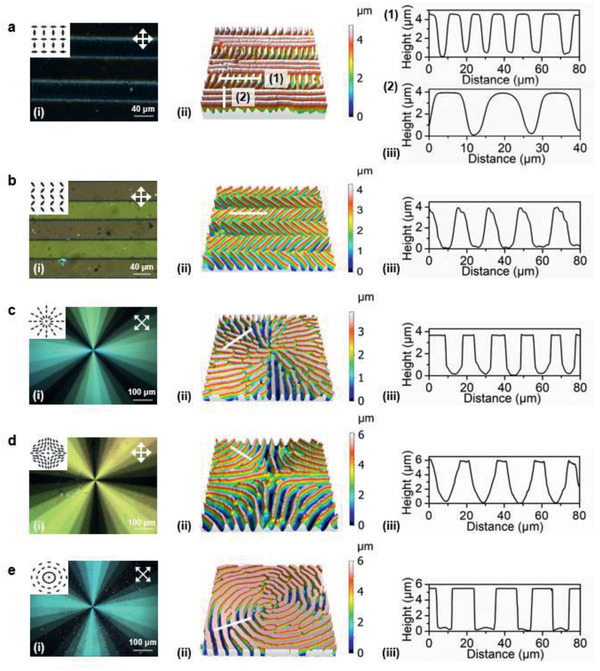
Formation of various complex topographies. a) Alternating horizontal and vertical stripes. b) Zigzag patterns. c) +1 radial defect. d) −1 defect. e) Azimuthal alignment. The images (i)–(iii) represent the POM image prior to actuation, the 3D surface topography after actuation and the corresponding 2D profile, respectively. The white line in the 3D topographies indicates the location where 2D profiles are extracted. The minor fluctuation in the periodicity is due to slightly different film thicknesses.

With the knowledge on that parallel oriented line patterns are generated from the anisotropic flow in LCP induced by the molecular alignment, we further advanced this system to create more complex surface topographic structures. In the first sample, we patterned adjacent rows of 40 µm wide with the molecular director $\hat{n}$ alternating orthogonal to each other as shown in **Figure** **4**a (the lines in the insert show the director pattern). The microscope image taken prior to the electrical actuation shows black between two crossed polarizers because of the alignment parallel to either one of the crossed polarizers. The bright thin lines in between relate to the orientational in‐plane transition between the two director states. Due to the elastic properties of the liquid crystal monomer, it needs a finite distance to transit from one orientation state to the other through a splay‐like configuration during which the molecules are under an angle with the crossed polarizers which consequently transmit light. After actuation, measured by 3D profilometer, topographies with alternating horizontal and vertical stripes that amplify the director patterns are formed (Figure 4a(ii),(iii)).

Similarly, we designed a zigzag pattern with a corner angle of 90° at their transition (Figure 4b(i)). POM image reveals birefringent color, in which the bright and dark yellow stripes imply slight asymmetry. Now, the transition area of $\hat{n}$ is the black line having the molecular director parallel to one of the polarizers. The actuated 3D protrusions and the corresponding 2D profile are given in Figure 4b(ii),(iii).

Next, we created a series of alignment structures around central defects. Figure 4c–e show a +1 radial defect, a −1 defect and an azimuthal alignment, respectively. Under the POM, they all exhibit fan‐shaped patterns as expected. The regions where molecules are parallel to one of the crossed polarizers appear black. By applying a DC field, 3D structures that replicate the director patterns are generated. Based on the actuation conditions, we varied the pitch and height of the formed topographies.

## 3 Conclusion

In conclusion, we developed a technique that stores latent information that can be retrieved repeatedly by subjecting the film to an electrical field. In its virgin state, the film is flat. But by applying an electrical field from an electrode in proximity a preset structure pops up. We have demonstrated this by creating various geometric figures, but it can be made as complex as needed for an application. The basic information is stored in an alignment layer by DMD technology and invisible by normal optical techniques. The information is optically amplified by the application of thin LCP film and mechanically amplified to its third dimension by electric development. This approach provides an alternative tool to create surface structures through the molecular self‐assembling process without involving tedious conventional lithographic processes. In addition, the morphing surfaces can alter a few macroscopic properties. Examples are, adhesion and release related tribology,^[^ ^27^ ^]^ which is relevant for robotic handling and haptic feedbacks; optical effects including dynamic scattering or reflection have drawn attention in the field of smart window applications;^[^ ^34^ ^]^ topographic profiles governed wettability for self‐cleaning function,^[^ ^35^ ^]^ and material transportation by breaking deformation symmetry.

## 4 Experimental Section

##### Materials

An overview of the materials is provided in Figure 1a. Monomers **1** and the crosslinker given in Figure 3d were obtained from HCCH (Jiangsu, China) and photoinitiator **2** from HEOWNS (Tianjin, China). Photo‐alignment agent (SD1) was obtained from Nanjing Murun New Material Technology Co., Ltd (Jiangsu, China). Typically, thin films were fabricated from a mixture containing monomer **1** and 1.0 w% photoinitiator **2**. In some experiments, photoinitiator **2** was used with different concentrations. The materials were mixed by dissolving in dichloromethane, which was evaporated at 50 °C subsequently. Homeotropically aligned coating was provided by an alignment layer provided homeotropic polyimide (Shenzhen Dalton Co., Ltd, DL 4018).

##### Sample Preparation

The cells for preparing liquid crystal film with uniaxial or patterned planar alignment were made using two indium tin oxide (ITO) coated glass substrates. SD1 photo‐alignment agent was coated via spin coating from 0.3 w% dimethylformamide (DMF) solution on top of the ITO electrode. The alignment layer was annealed at 100 °C for 5 min to completely remove DMF. The two substrates were connected to form the cell. The cell gap was chosen to be 3 µm by means of spherical spacers. DMD based dynamic microlithography system was used to scribe the desired alignment patterns in the cell. The LC mixture was filled into the cell by capillary force at 70 °C, the cell was cooled to 48 °C for 10 min. Photopolymerization was carried out by exposing the cell to UV light at an intensity of 25 mW cm^−2^ for 5 min, using an UV‐LED lamp (GZ LingKe Co.,Ltd, UVLED‐3030SKA). The typical modulus of the obtained material was 1 GPa. After polymerization, the top substrate was removed leaving the LCP film adhered to the bottom ITO glass substrate. A new cell for actuation purposes was made by mounting a plain ITO glass, without alignment layer, at a distance of 6 µm by means of spacers.

##### Characterization

The samples were actuated by a DC power source (Keithley 2400) which supplies voltages from 0 to 210 V. The optical properties of the LCP coating were characterized by polarized microscopy (POM; Leica DM2700P) equipped with a CCD camera (Infinity 1–3C) of 24‐bit resolution. The temperature of the devices was controlled by a thermal stage (Linkam THMS600). The thickness of the liquid crystal film and the surface profiles were measured Leica 3D profilometer in confocal mode (Leica DCM8). The mechanical properties of the film were tested by DMA (Mettler Toledo, DMA1‐247) at the frequency of 1 Hz with the applied force of 0.5 N. GIWAXS experiments were carried out on a GANESHA 300 XL+ system from JJ X‐ray equipped with a Pilatus 300K detector (pixel size 172 µm × 172 µm). The X‐ray source was a Genix 3D Microfocus sealed tube X‐ray Cu‐source with integrated monochromator and the wavelength used was *λ* = 1.5408 Å.

## Conflict of Interest

The authors declare no conflict of interest.

## Supporting information

Supporting Information

Supporting Information

Supporting Information
