# Supplementary material for: Translating 2D Director Profile to 3D Topography in a Liquid Crystal Polymer
Source: Adv Sci (Weinh). 2021 Feb 24;8(8):2004749. doi: 10.1002/advs.202004749 (PMC8061370; doi:10.1002/advs.202004749)
Supplement: Supplementary file 1 — Supporting Information [file ADVS-8-2004749-s003.pdf]

© 2020 Wiley-VCH GmbH

ADVANCED SCIENCE
Open Access

Supporting Information

for *Adv. Sci.,* DOI: 10.1002/advs.202004749

Translating 2D Director Profile to 3D Topography in a Liquid Crystal Polymer

Pengrong Lv, Yuxin You, Junyu Li, Yang Zhang, Dirk J. Broer, Jiawen Chen, Guofu Zhou, Wei Zhao and Danqing Liu**

Supporting information

Translating 2D Director Profile to 3D Topography in a Liquid Crystal Polymer

Pengrong Lv[1], *Yuxin You*[1], *Junyu Li*[2], *Yang Zhang*[3], *Dirk J. Broer*[1,4,5], *Jiawen Chen*[6], *Guofu Zhou*[1,6,7], *Wei Zhao*[1,6]* *and Danqing Liu*[1,4,5]*

[1] SCNU-TUE Joint Lab of Device Integrated Responsive Materials (DIRM), National Center for International Research on Green Optoelectronics, South China Normal University, No 378, West Waihuan Road, Guangzhou Higher Education Mega Center, 510006, Guangzhou China

[2] Molecular Materials and Nanosystems and Institute of Complex Molecular Systems, Eindhoven University of Technology, P.O. Box 513, Eindhoven 5600 MB, The Netherlands

[3] Solar Energy Research Institute, Yunnan Normal University, Kunming 650500, China

[4] Institute for Complex Molecular Systems, Eindhoven University of Technology, Den Dolech 2, 5612 AZ, Eindhoven, The Netherlands

[5] Department of Chemical Engineering and Chemistry, Eindhoven University of Technology Den Dolech 2, 5612 AZ, Eindhoven, The Netherlands

[6] Guangdong Provincial Key Laboratory of Optical Information Materials and Technology & Institute of Electronic Paper Displays, South China Academy of Advanced Optoelectronics, South China Normal University, Guangzhou 510006, P. R. China

[7] Shenzhen Guohua Optoelectronics Tech. Co. Ltd., Shenzhen 518110, China

*Corresponding author. Email: weizhao@m.scnu.edu.cn; d.liu1@tue.nl

Dielectric constant

The dielectric constant was calculated from the capacitance measured by an impedance analyzer. The measurement setup is illustrated in **Figure S1**a. Liquid crystal polymer films consisting of composite listed in Figure 1a with both planar alignment and homeotropic alignment were prepared. Top electrode was made by vacuum evaporation of a silver electrode on the LCP film. A 100 nm silver layer was formed at 35 ºC at the evaporation rate of 0.3 nm/s for 5.5 min. The dielectric constant can be calculated by equation: $\varepsilon_r = \frac{C\times d}{\varepsilon_0 S}$, where, C is measured capacitance d is film thickness, S is surface area, dielectric constant under vacuum ε_0=8.854×10^(−12) F/m.

For the planar aligned LCP film where the molecule long axis aligns parallel to the substrate, $\varepsilon_\perp$ is measured. While in case of a homeotropic film with the molecule long axis perpendicular to the substrate, $\varepsilon_\parallel$ is measured. Dielectric anisotropic is described by $\Delta\varepsilon = \varepsilon_\parallel - \varepsilon_\perp$, employing the results from Figure S1b, $\Delta\varepsilon$ takes a negative value.

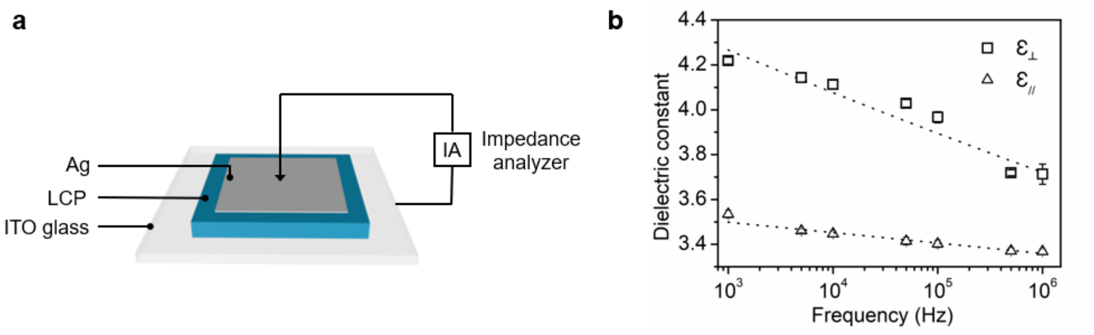

Figure S1. Dielectric constant measurement. a) Measurement setup. b) Dielectric response of LCP.

Differential scanning calorimetry (DSC)

We investigated the phase behavior of both the monomer and the polymer used in this work.

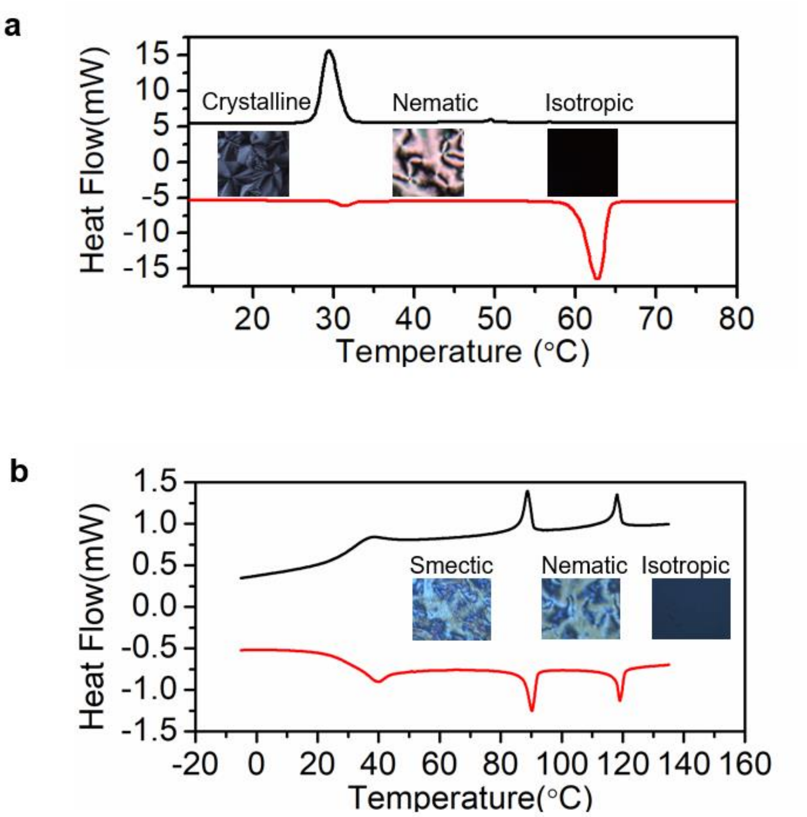

Figure S2. Thermal characterization of the materials. Differential scanning calorimetry (DSC) measurement (second run) shows the phase transition of the a) monomer mixture as indicated in Figure 1a, and b) its corresponding polymer. The measurement is carried out at the ramping rate of 5˚C/ min. The red curves are during heating; the black during cooling.

Dynamic mechanical analysis (DMA)

We characterized the mechanical properties of a freestanding polymer film. Its glass transition temperature analyzed from the tan delta maximum is approximately 46 ºC. At the actuating temperature of the film, 70 ºC, the polymer is in its molten state.

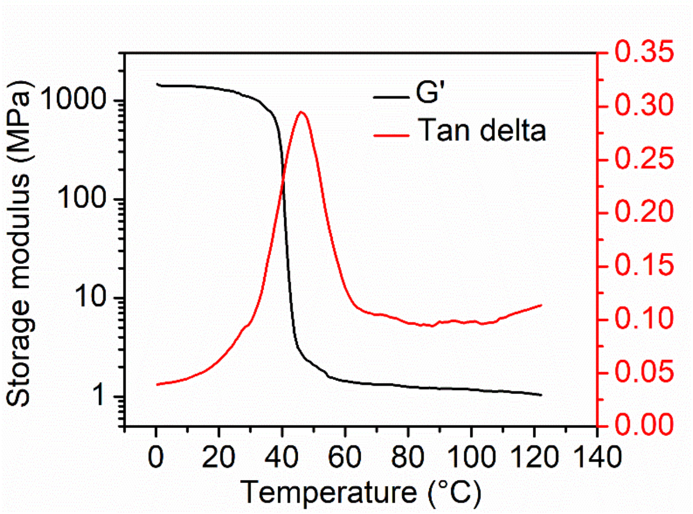

Figure S3. Storage modulus G` and tan delta as a function of temperature.

Formation of surface topographies at various molecular alignment

To estimate the influence of the phase on the formation of surface topographies, we actuated the coating at the smectic, nematic and isotropic state. The sample with smectic alignment (**Figure S4**a) has been described in the main text. The coating with planar nematic orientation, as shown in Figure S4b, exhibits line structures orthogonal to $\hat{n}$, as the polymer flows parallel to $\hat{n}$ as determined by the lowest viscosity in this direction. Sample shown in Figure S4c was actuated at 130 ºC, in the isotropic phase of the polymer. The resulting topographies were characteristic pillar patterns as currently also formed in reference isotropic polymers.

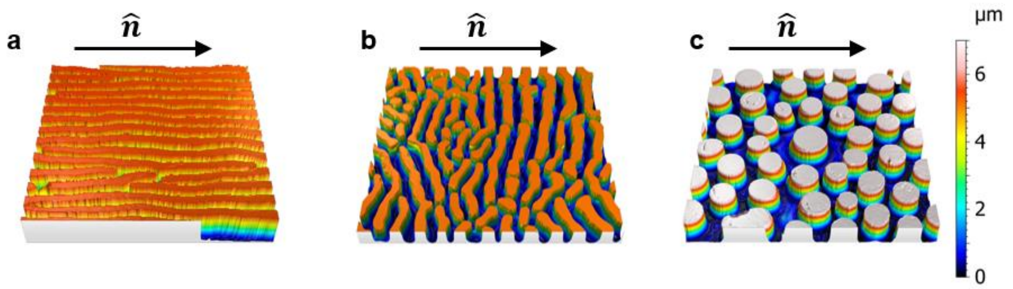

Figure S4. Influence of the molecular configurations on the formed surface topographies. Samples are actuated at a) smectic state at 70 ºC, b) nematic state at 93 ºC and c) isotropic state at 130ºC.

Thermal relaxation of the formed surface topographies

The formed surface topographic structures can relax back to its initial flat state thermally when the electric field is switched off while keeping the temperature at 70 ºC. This process takes 15 min, which can be further accelerated by heating the sample at an elevated temperature, for instance, at 100 ºC, the topographies are removed within 40 seconds. The dynamics of this process (at 70 ºC) is shown in Movie S2 of the Supporting Information.

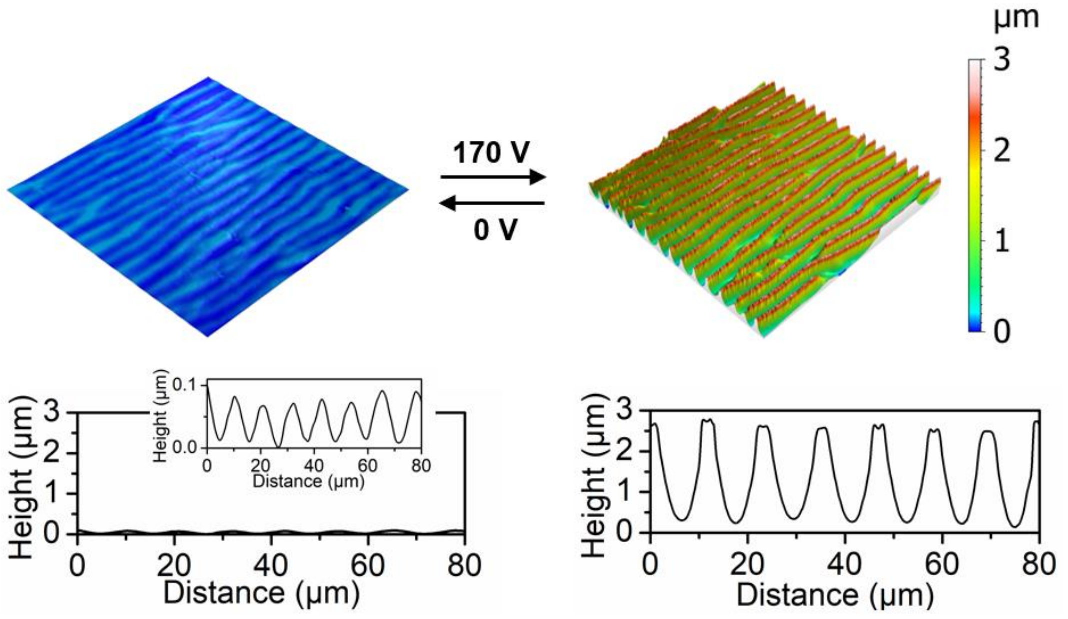

Figure S5. Thermal relaxation of the formed surface topographies.

Follow direction and the relation to the director during the structure formation

During the polymer flow process, the flow proceeds perpendicular to molecular director, which means parallel to the smectic layers. Therefore, in the final formed structures the molecular orientation is parallel to the longitudinal direction of the ridges.

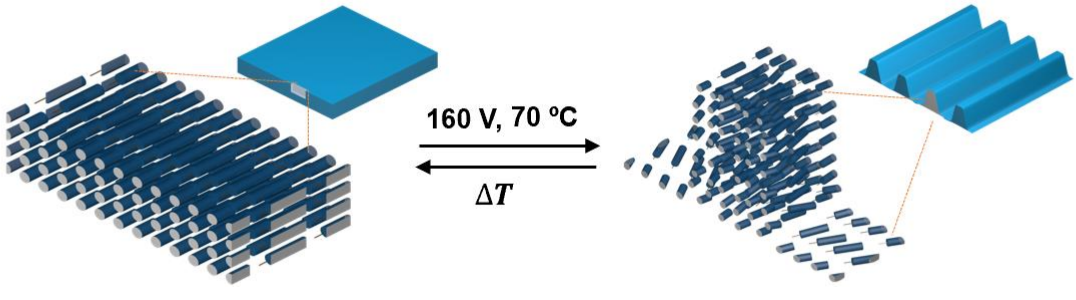

Figure S6. Schematic presentation of the molecular director.

Michel-Lévy Birefringence Chart

The initial optical retardation of the sample appears in the second order in the Michel-Lévy interference color chart, as indicated in **Figure S6**. The value is calculated as the product of film thickness of 3 μm and Δn which is approximately 0.19, estimated from its molecular structure (molecule **1**, Figure 1a). Upon actuation, the LCP melt flows from region **1** to region **2**, as indicated in Figure 2b-c, causing optical retardation of **1** transit to the first lower order, and finally towards zero, due to the thickness decreasing while that of **2** shifts to a higher order with increasing thickness.

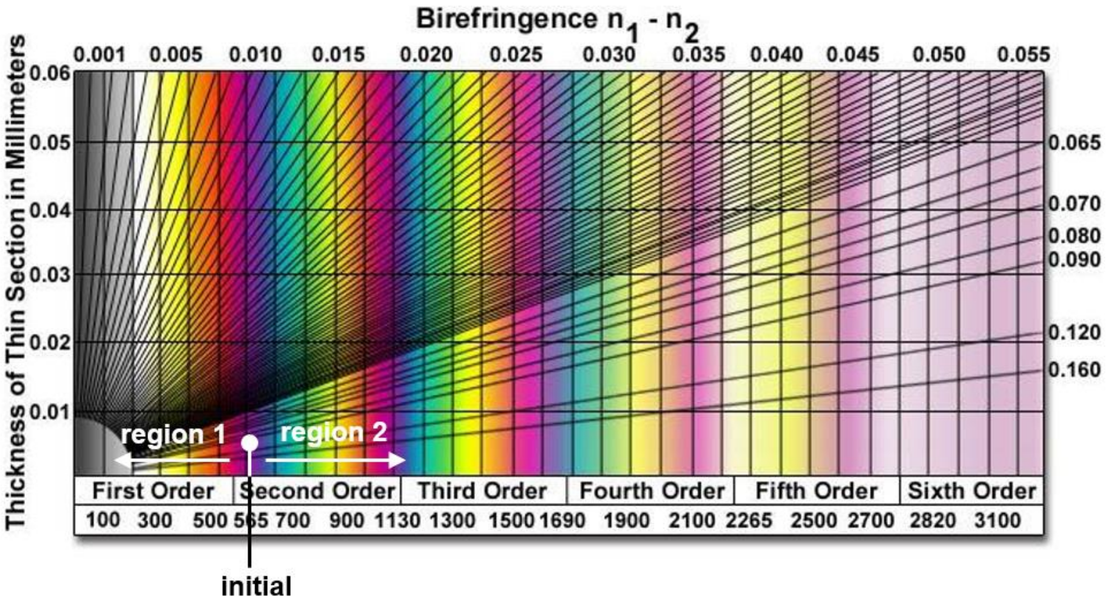

Figure S7. Correlating the birefringent color change during the formation of surface topographies with Michel-Lévy Birefringence Chart (edited from Olympus Microscopy[1]).

Using existing theories (for isotropic polymer) to understand the relationship between pitch and electric field strength in a quantitative way

According to the theory proposed by Schäffer et al. [2] for isotropic polymers, we can approximate the relationship between pitch and electric field strength, as described by the following equation.

$$\lambda = 2\pi \sqrt{\frac{\gamma U}{\varepsilon_p \varepsilon_0 (\varepsilon_p - 1)^2}} \left(\frac{U}{\varepsilon_p d - (\varepsilon_p - 1)h}\right)^{-\frac{3}{2}}$$

Where, $\boldsymbol{U}$ is the voltage applied across the cell. $\boldsymbol{\gamma}$ is the surface tension of the LCP which is measured to be about 29.33 mN/m at 70 °C. The spacing $\boldsymbol{d}$ between two electrodes is about 5.8 μm. The initial film thickness $\boldsymbol{h}$ is about 2.8 μm. The dielectric constant of the LCP $\boldsymbol{\varepsilon_p}$ (perpendicular to the electric field) is measured to be about 4.2 at 1000 Hz. The vacuum permittivity $\boldsymbol{\varepsilon_0}$ is 8. 854187817×10-12 F/m. The result is as shown below.

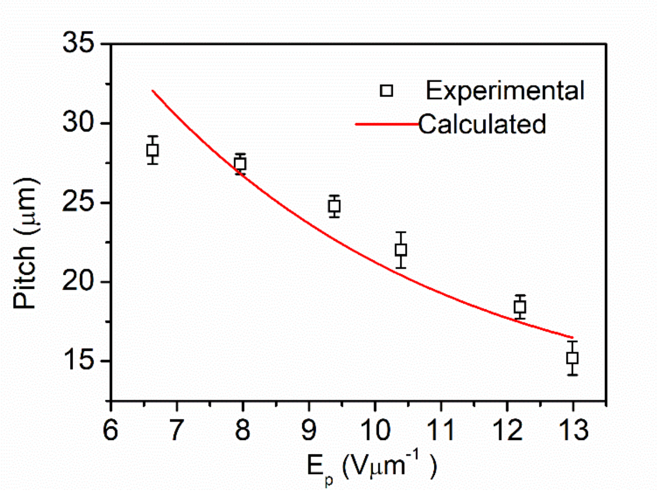

Figure S8. Measured and calculated relationship between pitch and electric field strength.

Determining polymer molecular weight by gel permeation chromatography (GPC)

The molecular weight of the liquid crystal polymer is estimated by GPC. During the test, the polymer was dissolved in tetrahydrofuran, using polystyrene as the calibrator. The test temperature was set at 25 °C and the flow rate was 1.0 mL/min.

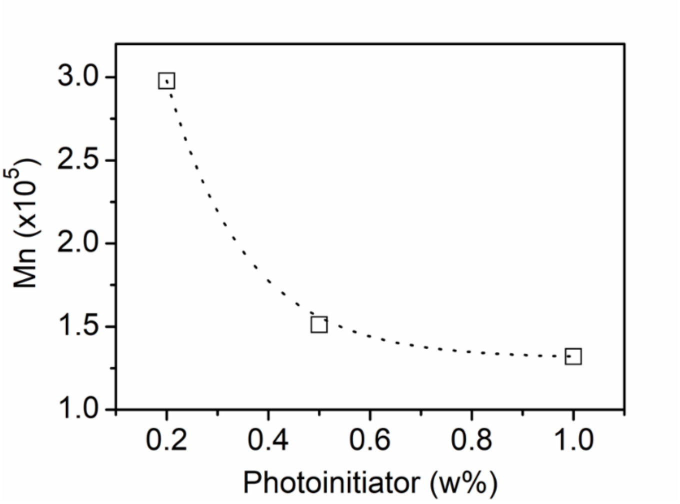

Figure S9. The molecular weight as the function of photoinitiator **2** concentration.

Movie S1-S2

Movie S1: The video 'Movie S1.mp4' shows the progress of protrusion formation. This realized via heating liquid crystal polymer film with uniaxial alignment to 70 ℃ and applying 160V at the same time.

Movie S2: The video 'Movie S2.mp4' shows thermal relaxation of the formed surface topographies. This realized via heating liquid crystal polymer film with planar alignment to 70 ℃ and applying 180V at the same time.

References

[1] M. W. D. Hoffman, Robert, "Specialized Microscopy Techniques - Michel-Levy Birefringence Chart | Olympus Life Science," can be found under https://www.olympus-lifescience.com/en/microscope-resource/primer/techniques/polarized/michel/, accessed: September, 2020.

[2] E. Schäffer, T. Thurn-Albrecht, T. P. Russell, U. Steiner, *Europhys. Lett.* **2001**, *53*, 518.
